# A New Method to Prepare Stable Polyaniline Dispersions for Highly Loaded Cathodes of All-Polymer Li-Ion Batteries

**DOI:** 10.3390/polym15112508

**Published:** 2023-05-29

**Authors:** Elena Tomšík, Daniil R. Nosov, Iryna Ivanko, Václav Pokorný, Magdalena Konefał, Zulfiya Černochová, Krzysztof Tadyszak, Daniel F. Schmidt, Alexander S. Shaplov

**Affiliations:** 1 Institute of Macromolecular Chemistry AS CR, Heyrovského Nám. 2, Prague 6, 162 00 Prague, Czech Republic; ivanko@imc.cas.cz (I.I.); pokorny@imc.cas.cz (V.P.); magdalenakonefal@imc.cas.cz (M.K.); cernochova@imc.cas.cz (Z.Č.); negaton83@gmail.com (K.T.); 2 Luxembourg Institute of Science and Technology (LIST), 5 Avenue des Hauts-Fourneaux, L-4362 Esch-sur-Alzette, Luxembourg; daniil.nosov@list.lu (D.R.N.); daniel.schmidt@list.lu (D.F.S.); alexander.shaplov@list.lu (A.S.S.); 3 Department of Physics and Materials Science, University of Luxembourg, 2 Avenue de l’Université, L-4365 Esch-sur-Alzette, Luxembourg

**Keywords:** polyaniline, acid-assisted synthesis, poly(ionic liquid), single-ion conductor, cathode material, 2D material, all-solid-state L batteries

## Abstract

A new method for the preparation of polyaniline (PANI) films that have a 2D structure and can record high active mass loading (up to 30 mg cm^−2^) via acid-assisted polymerization in the presence of concentrated formic acid was developed. This new approach represents a simple reaction pathway that proceeds quickly at room temperature in quantitative isolated yield with the absence of any byproducts and leads to the formation of a stable suspension that can be stored for a prolonged time without sedimentation. The observed stability was explained by two factors: (a) the small size of the obtained rod-like particles (50 nm) and (b) the change of the surface of colloidal PANI particles to a positively charged form by protonation with concentrated formic acid. The films cast from the concentrated suspension were composed of amorphous PANI chains assembled into 2D structures with nanofibrillar morphology. Such PANI films demonstrated fast and efficient diffusion of the ions in liquid electrolyte and showed a pair of revisable oxidation and reduction peaks in cyclic voltammetry. Furthermore, owing to the high mass loading, specific morphology, and porosity, the synthesized polyaniline film was impregnated by a single-ion conducting polyelectrolyte-poly(LiM_n_-*r*-PEGM_m_) and characterized as a novel lightweight all-polymeric cathode material for solid-state Li batteries by cyclic voltammetry and electrochemical impedance spectroscopy techniques.

## 1. Introduction

The role of lithium in the global economy is increasing as green energy is introduced. The acceleration of decarbonization leads to a high demand for lithium, with metal being the most important raw material for the production of modern electronics and electric vehicles (lithium-ion batteries and energy storage systems). With the economy recovering in 2021 and electric vehicle sales rising, the Li metal price has increased throughout the year and will remain high for the next decade. According to the U.S. Geological Survey (USGS) Mineral Commodity Summaries (MCS), although known lithium deposits have significantly increased in number worldwide, the general quantity of Li is limited and will be consumed by 2050 (for mineral ores) if the rate of consumption remains unchanged [1]. Thus, the decrease in amount of Li in batteries and the increase in capacity and gravimetric energy density remains crucial and represents an important topic for scientific studies [2,3].

Currently, the typical cathode materials used in commercial lithium ion batteries are mainly represented by inorganic compounds such as LiFePO_4_ (LFP) [4], LiCoO_2_ [5], LiNiO_2_ [6], LiNiMnCoO_2_ (NMC), etc. In spite of the benefits, all of them have drawbacks: relatively low specific capacity and poor electronic and ionic conductivity are known for LiFePO_4_ [7], LiCoO_2_ is very expensive, and LiNiO_2_ possesses a weak stability. One of the suggested solutions to solve some of the above-mentioned drawbacks was the application of electrochemically active electron-conducting polymers (EAECPs) into the LFP, NMC, etc., cathode structure to replace the conventional conductive carbons, improving the electronic conductivity and mechanical properties of cathode materials [7,8]. Later on, to decrease the Li amount in batteries, EAECPs were suggested to solely substitute the inorganic metal oxide cathode materials [9,10]. The theoretical values of conducting polymers specific capacity are comparable with those of metal oxide electrodes, while their synthesis is easier and significantly less expensive. Among semiconducting polymers, polyaniline (PANI) is uniquely attractive from an economic standpoint. The monomer is commercially available with a low price, the polymer can be synthesized via one step simple reactions, and the obtained PANI dispersions can be stabilized for storage and subsequent realization [11]. The most known method for PANI synthesis is the chemical oxidation of aniline by ammonium peroxydisulfate in the presence of organic or inorganic acids [12]. The perchloric acid-doped PANI nanotubes have already shown promising results when they were applied as sole cathode materials in lithium/polymer rechargeable batteries [13]. The charge–discharge curves demonstrated good reversibility with an average discharge–plateau voltage of 3.0 V and an average charge–plateau voltage of 3.4 V. The highest discharge capacity of PANI nanotubes/nanofibers-based Li batteries reached 75.7 mA h g^−1^ out of 147 mA h g^−1^ theoretically calculated capacity [13]. However, such loss in capacity and unsatisfactory performance of neat PANI as a cathode material explained the strong agglomeration of the PANI chains, and the inaccessibility of interior of the resultant nanotubes with respect to ion intercalation/penetration [14]. Therefore, the modification of the PANI synthesis and its molecular self-assembly at the cathode is desirable.

Recently, our group [15,16], in parallel with the team of George Chen [16], demonstrated that PANI chains could be specifically assembled in 3D structures with 80% crystallinity by chemical polymerization of aniline in the presence of Hoffmeister ions. The intercalation of ions into such PANI 3D structures occurs much faster compared to the agglomerated state of common PANI. However, the limitation of this method was the mass loading of PANI that can be achieved on a current collector. The simple layer deposition resulted in the formation of thin films (~100–200 nm) that demonstrated excellent results in sensor applications [17]. However, the attempts to increase the thickness of the PANI layer via layer-by-layer deposition led to only 1–5 mg cm^−2^ of PANI mass loading [15]. A maximum of 2.4 mg cm^−2^ of PANI mass loading on the conductive carbon cloth electrode was realized after multiple sequential polymerization deposition steps [16,18,19].

**Scheme 1:**
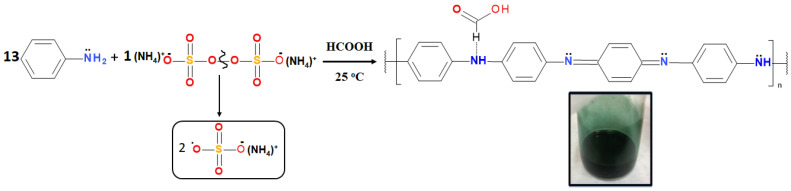
Acid-assisted polymerization of aniline in the presence of formic acid.

In the present study, we report a very simple and clean one-step reaction route for the synthesis of a stable PANI suspension (Scheme 1), whose deposition on fluorine-doped tin oxide-coated (FTO) glass or conductive carbon cloth (CC) current collectors lead to a *never-before-achieved record high mass loading of electro-active material* (30 mg cm^−2^). The resulting PANI film possesses a 2D structure, as confirmed by X-ray diffraction measurements, and shows significant porosity, as observed via SEM. This structure was found to be ideal for impregnation by solutions of single ion conducting polyelectrolytes (SPEs), leading to the formation of an attractive composite cathode material and facilitating ion intercalation during charging and discharging processes.

## 2. Materials and Methods

### 2.1. Materials

Formic acid (>88%, Aldrich, St. Louis, MO, USA), ammonium peroxydisulfate (99%, Lach-ner, Neratovice, The Czech Republic), ethyl alcohol (99.8%, Aldrich), aniline (99+%, analytic grade, Lach-ner), poly(ethylene glycol)methyl ether methacrylate (PEGM, M_n_ = 500 g/mol, Aldrich), 3-sulfopropyl methacrylate potassium salt (98%, Aldrich), lithium hydride (LiH, 97%, Sigma-Aldrich), trifluoromethanesulfonamide (97%, ABCR, Karlsruhe, Germany), triethylamine (TEA, Et_3_N, ≥99%, Merck, Rahway, NJ, USA), lithium bis(trifluoromethylsulfonyl)imide (99+%, ABCR), 4-cyano-4-(phenylcarbonothioylthio)pentanoic acid (CPAD, chain transfer agent (CTA), >97%, Aldrich), dichloromethane (DCM, 99.8%, Aldrich), dimethylformamide (DMF, anhydrous, 99.5%, Acros, Waltham, MA, USA), N-methyl-2-pyrrolidone (NMP, anhydrous, 99.5%, Acros), diethyl ether (99%, Aldrich), methanol (99.8%, Aldrich), and tetrahydrofuran (THF, anhydrous, Acros) were used as received. Thionyl chloride (99.7% Acros) was distilled over linseed oil. 2,2′-Azobisisobutyronitrile (AIBN, initiator, 98%, Acros) was recrystallized from methanol. 4-Methoxyphenol (99%, Acros) was sublimed in a vacuum prior to use.

Fluorine-doped tin oxide-coated glass slides (FTO, SnO_2_/F, Aldrich, 100 × 100 × 2.2 mm^3^, surface resistivity 7 Ω/sq) were placed in an ultrasonic bath with acetone for 15 min, then with ethanol for 15 min and with water for 15 min. The slides were dried with N_2_ flow and in vacuum oven at 60 °C/1 mbar for 4 h before polyaniline coating. Carbon cloth (CC, Elat^®^, Fuel Cells Etc. Co., Bryan, TX, USA, ELAT-hydrophilic plain cloth, 3 × 4 cm^2^, 13 mg cm^−2^ 80% porosity, 99.5% carbon content) was washed with ethanol in ultrasonic bath for 15 min, then with distilled water and dried in a vacuum oven at 60 °C/1 mbar for 4 h before **PANI-a** and **PANI-c** deposition.

### 2.2. Synthesis of Polyaniline via Acid-Assisted Polymerization (**PANI-a**)

Acid-assisted polymerization was carried out in one step in the presence of ammonium peroxydisulfate following the procedure described below. Aniline (0.510 g, 0.50 mL, 5.5 mmol) was dissolved at room temperature in 4.7 mL of concentrated formic acid and 1 mL of ethanol. Ammonium peroxydisulfate (0.094 g, 0.4 mmol) was dissolved in 1 mL of distilled water and was added dropwise into the solution of aniline. The molar ratio of monomer (aniline) to oxidant was 1.0:0.076. The polymerization was continued at room temperature for 48 h and UV-VIS spectroscopy was used to determine the completeness of the reaction (absence of monomer). After the evaporation of formic acid and ethanol, polyaniline (**PANI-a**) was obtained as a dark green powder. Polymer was washed with ethanol and the product was dried at 60 °C/1 mbar for 10 h. Yield: 0.522 g (99%, with protonation by HCOOH equal to 5%); Raman (785 nm laser): 1590, 1500, 1350, 1260, 1175 cm^−1^; σ = 0.41 ± 0.02 S cm^−1^ (25 °C).

The same procedure was repeated for the deposition of **PANI-a** film on the current collector. After completeness of polymerization, the resulting PANI dispersion was directly deposited either on the surface of the FTO glass slide or on CC with slow (!) solvent evaporation at room temperature to form a thin **PANI-a** film. The resultant film was rinsed with ethanol (to remove the oxidant residue) and dried at 60 °C for 2 h. For electrochemical characterization the total amount of **PANI-a** was 60 mg, which corresponds to 30 mg cm^−2^.

### 2.3. Synthesis of Polyaniline via Common Chemical Polymerization (**PANI-c**)

Polyaniline (**PANI-c**) was prepared in accordance with the previously published method [16]. In brief, aniline (0.10 mL, 0.001 mol, 0.04 mol L^−1^) was dissolved in a 12.5 mL of 5 M aqueous formic acid mixed with NaCl (6.092 g, 6.4 mol L^−1^). The aniline solution was cooled down to 5 °C in the ice bath and the solution of ammonium peroxydisulfate (0.224 g, 0.41 mmol, 0.04 mol L^−1^) in 12.5 mL of 5 M formic acid mixed with NaCl (6.092 g, 6.4 mol/L) was added to it dropwise. The total volume of the polymerization solution reached 25 mL. The reaction continued at 5 °C for 60 min, whereupon the obtained dispersion was deposited on FTO or CC surface. To obtain the film with active mass loading of **PANI-c** equal to 2.5 mg cm^−2^, four sequential polymerization reactions were carried out (4 PANI layers were deposited). The resultant film was rinsed with ethanol (to remove the oxidant residue) and dried at 60 °C for 2 h.

### 2.4. Synthesis of Lithium 1-[3-(methacryloyloxy)propylsulfonyl]-1-(trifluoromethanesulfonyl)imide (LiM)

Lithium 1-[3-(methacryloyloxy)propylsulfonyl]-1-(trifluoromethanesulfonyl)imide was synthesized in accordance with the procedures published previously [20,21]. The monomer was crystallized from anhydrous dichloromethane, and the resultant crystalline powder was dried at 25 °C/1 mbar overnight and stored under inert atmosphere in an argon-filled glove-box (MBRAUN MB-Labstar, H_2_O and O_2_ content < 0.5 ppm). Spectroscopic data of the target compound were in full accordance with those reported in the literature [22].

### 2.5. Synthesis of Random Poly(LiM_13_-r-PEGM_81_) Copolymer

Random copolymer poly(LiM_13_-*r*-PEGM_81_) was prepared via controlled RAFT copolymerization following the procedure given below.

PEGM (14.48 g, 28.97 mmol) and LiM (2.00 g, 5.79 mmol) were dissolved in 52.4 mL (49.45 g) of anhydrous DMF at room temperature under inert atmosphere, whereupon the AIBN (11.50 mg, 70.15 μmol) and CPAD (98.00 mg, 350.73 μmol) were added, and the stirring was continued until the formation of a clear pink solution. The following initial reagent ratios were loaded: ([PEGM]_0_ + [LiM]_0_):[CPAD]_0_:[AIBN]_0_ = 99.1:1:0.2. The solution was quantitatively transferred to the Schlenk flask equipped with magnetic stirring bar, degassed by three freeze–evacuate–thaw cycles, flashed with argon, and placed in the bath preheated to 60 °C. Polymerization was continued at 60 °C for 44 h. The resultant viscous solution was cooled down to RT, diluted with DMF, and precipitated into the excess of diethyl ether. Copolymer was collected, redissolved in acetone with the addition of 4-methoxyphenol (polymerization inhibitor, 40 mg), and precipitated for the second time into the excess of diethyl ether. The product in form of a pink viscous cold flowing liquid was collected and dried at 55 °C/0.1 mbar for 12 h in B-585 oven (Buchi Glass Drying Oven, Flawil, Switzerland) and filled with P_2_O_5_. Yield: 13.30 g (81.0%). The total conversion determined by ^1^H NMR: q = 0.96. The ratio between LiM and PEGM in the copolymer was determined by ^1^H NMR as 1:6.2 by mol; ^1^H NMR (600 MHz, DMSO-d_6_): δ = 3.89–4.20 (t, 14H, -CO-O-CH_2_), 3.38–3.87 (m, 212H, -CH_2_-O-CH_2_-CH_2_-O-), 3.24 (s, 18.6H, -O-CH_3_), 3.00 (s, 2H, -CH_2_-SO_2_-N-SO_2_CF_3_), 1.25–2.20 (m, 16H, -CH_2_-CH_2_-SO_2_-N-SO_2_CF_3_, -CH_2_-C(CH_3_)), 0.50–1.24 (m, 22H, -CH_2_-C(CH_3_)); ^19^F NMR (565 MHz, DMSO-d_6_): δ = −79.8 (s, CF_3_); ^7^Li NMR (233.2 MHz, DMSO-d_6_): δ = −1.03 (s, Li); IR (ATR-mode): 2947 (m, ν_C-H_), 2872 (s, ν_C-H_), 1723 (vs, ν_C=O_), 1475 (m), 1451(m), 1404 (m), 1379 (w), 1352 (m, ν_asSO2_), 1326 (w), 1265 (m), 1246 (m), 1227 (m), 1179 (s, ν_CF_), 1105 (vs, ν_asC-O-C_), 1055 (s, ν_CF_), 947 (m), 858 (s, νs_C-O-C_), 842 (s), 789 (w), 749 (w) cm^−1^; *M*_n(SEC)_ = 43.7 kDa, *M*_w_/*M*_n(SEC)_ =1.53; *T*_g_ = −53 °C (DSC 5 °C min^−1^); *T*_onset_ = 150 °C (TGA, 5 °C min^−1^, on air); σ_DC_ = 4.1 × 10^−7^ S cm^−1^ (25 °C).

### 2.6. Preparation of SPE/PANI-a@CC Composite Polymer Cathode

The coating of **PANI-a** deposited on carbon cloth (hereinafter abbreviated as **PANI-a@CC**), as described in Section 2.2., was used for further preparation of the composite cathode by its uniform impregnation with the 0.1 wt.% solution of poly(LiM_13_-*r*-PEGM_81_) in anhydrous NMP. The poly(LiM_13_-*r*-PEGM_81_) solution was filtered through 0.22 μm syringe filter and cast at room temperature on top of **PANI-a@CC** placed on the leveled hotplate, whereupon the surface of the hotplate was heated to 70 °C. The as-obtained cathode tape was further dried in an oven at 70 °C/1 mbar for 24 h.

### 2.7. Measurements

NMR spectra were recorded on Avance III HD 600 MHz spectrometer (Bruker, Mannheim, Germany) at 25 °C in the indicated deuterated solvent and are listed in ppm. The signals corresponding to the residual protons and carbons of the deuterated solvent were used as an internal standard for ^1^H and ^13^C NMR, respectively. The C_6_F_6_ (−164.9 ppm) and LiBF_4_ (−1.04 ppm) were utilized as external standards for ^19^F and ^7^Li NMR, correspondingly. Raman spectra were registered on an InVia™ microspectrometer (Renishaw plc, Wotton-under-Edge, UK) equipped with a DM LM microscope (Leica microsystems, Wetzlar, Germany). The spectra were measured with two excitation lasers: Ag laser (514 nm excitation) and NIR diode laser (785 nm excitation) with gratings 2400 and 1200 lines mm^–1^, respectively. IR spectra were acquired on an INVENIO-R Fourier-transform IR-spectrometer (Bruker, Germany) using ATR technology with 128 scans and a resolution of 2 cm^−1^. The electron paramagnetic resonance (EPR) measurements were conducted with ELEXSYS 540 X band spectrometer (Bruker, Germany) equipped with 049X microwave bridge (Super X) and ER4108 TMHS slim design resonator (Bruker, Germany). The following EPR spectrometer settings were applied for the experiments: 6.362 mW (15 dB) microwave power; 0.1 G (L-3) and 1 G (M-10) modulation amplitude; 100 kHz frequency modulation; 5.12 ms time constant; 20.48 ms conversion time; 60 dB gain; resolution of 2048 points and at temperature of 295 K. G-factors were estimated by comparison with DPPH free radical standard, assuming that g-factor is equal to 2.0036. Line asymmetry was defined as the ratio of positive amplitude (A) to the absolute value of the negative amplitude (|B|) of the first derivative of resonant microwave absorption recorded in EPR.

The size exclusion chromatography (SEC) was used to determine the number-average molecular weight (*M*_n_(SEC)) and *M*_w_/*M*_n_ ratio for ionic random poly(LiM_13_-*r*-PEGM_81_). The study was performed on a 1200 Infinity gel permeation chromatograph (Agilent Technologies, Santa Clara, CA, USA) equipped with PLgel 5 μm MIXED-D column (Agilent Technologies, USA), PLgel 5 μm (Agilent Technologies) pre-column and an integrated refractive index detector. The system was operated at 50 °C and 1.0 mL/min flow using 0.1 M Li(CF_3_SO_2_)_2_N solution in DMF as an eluent. Poly(methyl methacrylate) standards (EasiVial PM, Agilent Technologies, *M*_p_ = 550–1558 × 10^3^) were used to perform calibration.

Thermogravimetric analysis (TGA) was carried out in air on a TGA2 STAR^e^ System (Mettler Toledo, Greifensee, Switzerland) by applying a heating rate of 5 °C min^−1^. The onset weight loss temperature (*T*_onset_) was determined as the point in the TGA curve at which a significant deviation from the horizontal was observed. The resulting temperature was then rounded to the nearest 1 °C. For DSC measurements, the polymer sample was preliminary dried at 60 °C/1 mm Hg for 12 h in the B-585 oven (Buchi Glass Drying Oven, Switzerland), filled with P_2_O_5_, and was transferred under a vacuum inside an argon-filled glovebox (MBRAUN MB-Labstar, H_2_O and O_2_ content < 0.5 ppm), where it was hermetically sealed in Al pans. Prior to measurements, the calorimeter was calibrated using the indium calibration standard (Mettler-Toledo, purity > 99.999%). The DSC of polymer sample was performed on a DSC3^+^ STAR^e^ System (Mettler Toledo, Switzerland) with a heating rate of 5 °C min^−1^ (1st and 2nd cycles) and 10 °C min^−1^ (3rd cycle) in the range of −80 to 80 °C. The glass transition temperature (*T*_g_) was determined during the second heating cycle, as the first one was used to eliminate the thermal history of the sample.

Diffraction patterns were obtained using a high-resolution diffractometer Explorer (GNR Analytical Instruments, Novara, Italy) equipped with a 1D silicon strip detector Mythen 1K (Dectris, Baden, Switzerland). A sealed X-ray tube operated at 40 kV, 35 mA, and monochromatized with Ni foil (β filter) was used to produce CuKα radiation (wavelength λ = 1.54 Å). Measurements were performed in the range 2θ = 2–35° with 0.1° step and an exposure time of 10 s at each step.

Static and Dynamic Light Scattering (SLS and DLS). The intensity-weighted hydrodynamic radius (*R*_H_) and scattering intensity of the **PANI-a** samples in the form of formic acid solutions were studied at 20 °C as a function of scattering angle using ALV-6000 equipment (ALV-GmbH, Langen, Germany). The data were processed with the Repes algorithm [23]. The weight average molecular mass ($\bar{M_{w}}$) and radii of gyration ($\bar{R_{g}}$) for PANI particles were determined at 20 °C by SLS method using the same ALV-6000 equipment. The z-average radii of gyration of particles ($\bar{R_{g}}$) and their *M*_w_ were calculated from a wide range of scattering angles (20° to 150°, increment 2°) and polymer concentrations (6.0, 5.45, 5.0, and 4.5 µg mL^−1^ in the solution of formic acid). All solutions were filtered using 0.46 µm PVDF syringe filter prior investigations. The SLS was measured three times and the SLS acquisition time was 90 s. Subsequently, the Guinier correlations were plotted from the obtained data and analyzed by the ALV/static and Dynamic FIT and PLOT 4.31 10/01 software ((ALV-GmbH, Germany).

Electrochemical impedance spectroscopy (EIS) was applied to determine the ionic conductivity (σ_DC_) of poly(LiM_13_-*r*-PEGM_81_) copolymer using a VSP potentiostat/galvanostat (Bio-Logic Science Instruments, Seyssinet-Pariset, France). To avoid any influence of moisture/humidity on the conductivity of polymer electrolyte, the latter was preliminary dried at 60 °C/1 mbar for 12 h in the B-585 oven (Buchi Glass Drying Oven, Switzerland) filled with P_2_O_5_ and transferred under a vacuum inside an argon-filled glovebox (MBRAUN MB-Labstar, H_2_O, and O_2_ content < 0.5 ppm). The polymer was sandwiched between two stainless steel (SS-316) blocking electrodes. The distance between the electrodes was kept equal to 250 µm using a Teflon spacer ring with the inner area of 0.502 cm^2^. The symmetrical stainless steel/copolymer/stainless steel assembly was clamped into the 2032 coin cell and was then taken out from the glovebox. Cell impedance was measured at the open circuit potential (OCP) by applying a 10 mV perturbation in the frequency range from 10^−2^ to 2 × 10^5^ Hz and in a temperature range from 20 to 100 °C. Temperature was controlled using the programmed KT-53 oven (Binder, Tuttlingen, Germany), where cells were allowed to reach thermal equilibrium for at least 1 h before each test.

Electrochemical characterization of the neat electrodes (**PANI-a@CC** and **PANI-c@CC**) was carried out by cyclic voltammetry (CV) in 0.2 M aq. HCl liquid electrolyte. The CV was performed at 25 °C in three electrodes cell configuration using an AUTOLAB PGSTAT302N potentiostat (Metrohm Inula GmbH, Prague, Czech Republic) with a FRA32M Module and Nova 2.1 software. A Pt sheet (1.2 cm^2^) was used as the counter electrode, and the Ag/AgCl wire was applied as the pseudo-reference electrode. The potential sweeps were carried out between −0.1 and 0.8 V vs. Ag^+^/Ag at variable scan rates.

The CV of **SPE/PANI-a@CC** composite cathodes was performed in both liquid electrolyte and in a solid state. (1) The CV in liquid electrolyte was recorded using the three-electrode cell (CC as working, Pt foil as counter, and Ag/AgCl wire as the reference electrodes) in 2.0 M lithium bis(trifluoromethylsulfonyl)imide (LiTFSI) solution in a mixture of ethylene carbonate/dimethyl carbonate with a ratio of 1:1 by volume. The CV was measured at 25 °C on AUTOLAB PGSTAT302N potentiostat (Metrohm Inula GmbH, Czech Republic) in the potential window ranging from −0.9 to 1.0 V vs. Ag^+^/Ag with various scan rates. (2) For the CV in solid state, the Li||**SPE/PANI-a@CC**||Cu cell was assembled inside the glovebox (MBRAUN MB-Labstar, H_2_O and O_2_ content < 0.5 ppm). Li foil was used as a reference and counter electrode, and **SPE/PANI-a@CC** was used as a working electrode. The hermetically sealed cell was taken out of the glovebox, placed in the heating oven for 1 h at 70 °C for conditioning, and then tested by cyclic voltammetry applying 1 mV s^−1^ sweep rate at 70 °C. Five continuous cycles were performed and recorded.

The EIS for **SPE/PANI-a@CC** composite cathodes were measured in liquid electrolyte and in a solid state on AUTOLAB PGSTAT302N potentiostat (Metrohm Inula GmbH, Czech Republic), applying the frequency range from 10^−1^ to 10^−4^ Hz with 5 mV perturbation. All measurements were performed at an open circuit potential (OCP). (1) The EIS in liquid electrolyte was carried out at 25 °C using 2.0 M LiTFSI solution in a mixture of ethylene carbonate/dimethyl carbonate with a ratio of 1:1 by volume. (2) For the EIS in solid state, the **SPE/PANI-a@CC** cathode was sandwiched between two stainless steel (SS-316) blocking electrodes inside an argon-filled glovebox (MBRAUN MB-Labstar, H_2_O, and O_2_ content < 0.5 ppm). The distance between the electrodes was kept equal to 250 µm using a Teflon spacer ring with the inner area of 0.502 cm^2^. The symmetrical stainless steel||**SPE/PANI-a@CC**||stainless steel assembly was clamped into a 2032 coin cell and was taken out from glovebox afterwards. The EIS was further measured at 25 and 70 °C. To reach the equilibrium, the corresponding temperature was applied for 2 h before the measurement.

## 3. Results and Discussions

### 3.1. Synthesis of Polyaniline via Formic Acid-Assisted Polymerization (**PANI-a**)

Several synthetic techniques have been applied to obtain organized PANI film for different applications (sensors, electro-magnetic shielding, pseudo-capacitors, and batteries). These techniques include chemical oxidation, electrochemical polymerization [24], template-assisted chemical method [25], interface polymerization [26], etc. The chemical oxidation of aniline has been conducted in the presence of various dopant acids (hydrochloric acid (HCl) [27,28], sulfuric acid (H_2_SO_4_) [29,30], o-amino benzoic acid [31], dodecylbenzene sulfonic acid [32], formic acid [33], acetic acid (AA) [30], malic acid (MA) [34], propionic acid (PA) [34], succinic acid (SA) [34], tartaric acid (TA) [34], citric acid (CA) [34], (etc.) and oxidants (ammonium persulfate (APS), vanadic acid [35], hydrogen peroxide (H_2_O_2_) [36], potassium dichromate (K_2_Cr_2_O_7_) [37], iron(III) chloride (FeCl_3_) [38], potassium permanganate (KMnO_4_) [39], etc.). The nature of the acid used is believed to be of high importance, as it simultaneously acts as a dopant and as a structure-directing agent. Both the acidity of the medium and its concentration affect the morphology of the synthesized PANI, varying from flowers, sheets [27], and plates [40] to nanotubes [34,41], nanorodes [35], and nanofibers [27]. In contrast to oxidative chemical polymerization, where the ratio of aniline monomer: ammonium peroxydisulfate is **1:1** or **1:1.25** [41], the acid-assisted aniline polymerization is suggested to be a radical process, which was proved by measuring EPR signal for freshly mixed polymerization solution (SI, Figure S1). Although the acid-assisted polymerization of aniline has been known (see [42,43,44]), here we report a significantly improved method with the utilization of concentrated formic acid, proceeding at room temperature without the need for cooling. The process consists of the addition of aqueous ammonium peroxydisulfate solution to the solution of aniline in the mixture of concentrated formic acid and ethanol (Scheme 1). The optimization of the process allowed to us establish the optimal monomer/initiator ratio equal to **13:1**, leading to the formation of a stable PANI suspension. The video of the synthetic process is shown in the Supporting Information (Video S1). From the initiation centers the reaction starts with the formation of dark blue colored oligomers, and 30 min at ambient temperature results in the dark green PANI suspension (Figure S1 and Video S1). It was found that the concentration of the formic acid plays a crucial role for the stability of the suspension due to the interaction with polymer chains via protonation as it was previously shown for another polymer-poly (3,4-ethylene dioxythiophene) (PEDOT) [44]. The concentration of the PANI in suspension was only limited by the amount of added monomer. With the increase in aniline concentration, the reaction proceeded faster.

The obtained PANI suspension was very stable without any precipitation for at least six months and the PANI mass loading on the current collector surface could be further controlled by changing either the deposition volume of the suspension and/or by dilution of the suspension with concentrated formic acid or any other organic solvent with the exception for water, which leads to the immediate coagulation (precipitation) of PANI.

### 3.2. Study of **PANI-a** Suspension via Dynamic (DLS) and Static Light Scattering (SLS)

**Figure 1:**
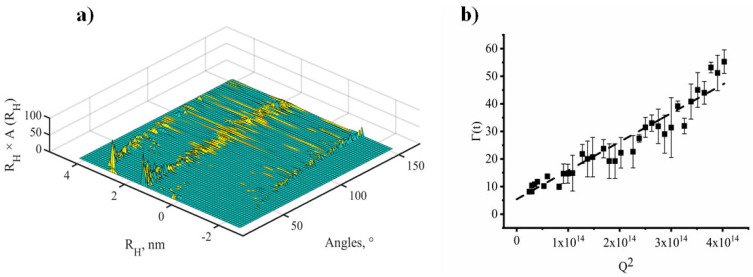
(**a**) Angular dependence of PANI-a 6 µg/mL in formic acid solution at room temperature; (**b**) Decay rate vs. square scattering vector dependence for PANI sample with a concentration of 6 mg mL^−1^ in formic acid.

The **PANI-a** suspension was studied via dynamic (DLS) and static light scattering (SLS) analyses. During DLS measurements of **PANI-a** colloid suspension at different angles, structures with a hydrodynamic radius of 50 nm were detected. The distribution of their sizes depicted as a 3D diagram (Figure 1a) demonstrated unimodal character. 

The equal-area representation (the Z-axis shows R_H_·A(R_H_), where A(R_H_) is the intensity-weighed distribution function of hydrodynamic radii R_H_ of particles in the sample) was applied for plotting Figure 1a. The results indicated that the 50 nm **PANI-a** particles were fairly stable in terms of colloidal homogeneity, showing practically no coalescence and sedimentation. The dependence of decay rate Γ vs. the square of the scattering vector q (Figure 1b) demonstrated linear character and approached above zero when crossing the y-axis. This can serve as proof of the rotational diffusion of these particles. The results of SLS and DLS experiments are presented in Table 1. The value of *v = R_H_/R_g_* = 1.718 can be ascribed to the elongated rod-like shape of the particles similar to those rod-type structures observed for block copolymers obtained from N-(2-hydroxypropyl)-methacrylamide and N-(2N′-Boc-aminoethyl)acrylamide in aqueous solution [45]. The key role of the presented new synthetic method for **PANI-a** synthesis is the possibility to obtain stable PANI suspension with high concentration and without addition of any soluble polyelectrolyte. The observed stability can be explained by two factors: (a) the small size of the obtained rod-like particles (50 nm) and (b) the change of the surface of colloidal PANI particles to a positively charged form by protonation with concentrated formic acid. These conclusions can be additionally proved by comparison with **PANI-c** synthesis, where the concentration of the weak formic acid is not enough to efficiently modify the PANI surface charge, and the agglomeration and sedimentation of the particles are observed within a few minutes from the start of reaction.

### 3.3. **PANI-a** Physical and Electrochemical Properties

**Scheme 2:**
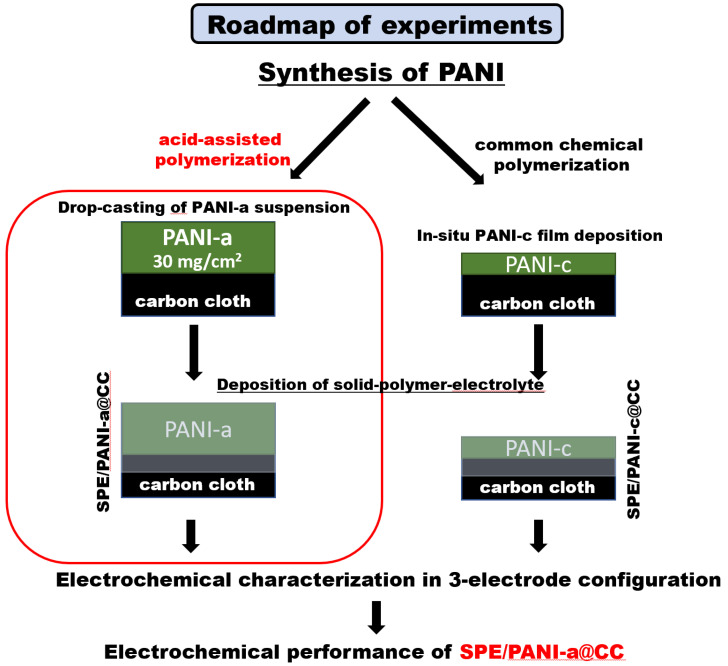
Roadmap of the experimental part.

The newly obtained PANI suspension (denoted as **PANI-a**) and coatings on FTO and CC (herein and after-mentioned as (**PANI-a@FTO** and **PANI-a@CC**) were characterized by a set of methods and further compared with the PANI prepared by “common” oxidation method (denoted as **PANI-c**). The “Roadmap” of the experiments is shown in Scheme 2. It must be emphasized that **PANI-c** has been reported in the literature [16] and is shown in the current study only for comparison purposes.

#### 3.3.1. Spectroscopic Characterization

**Figure 2:**
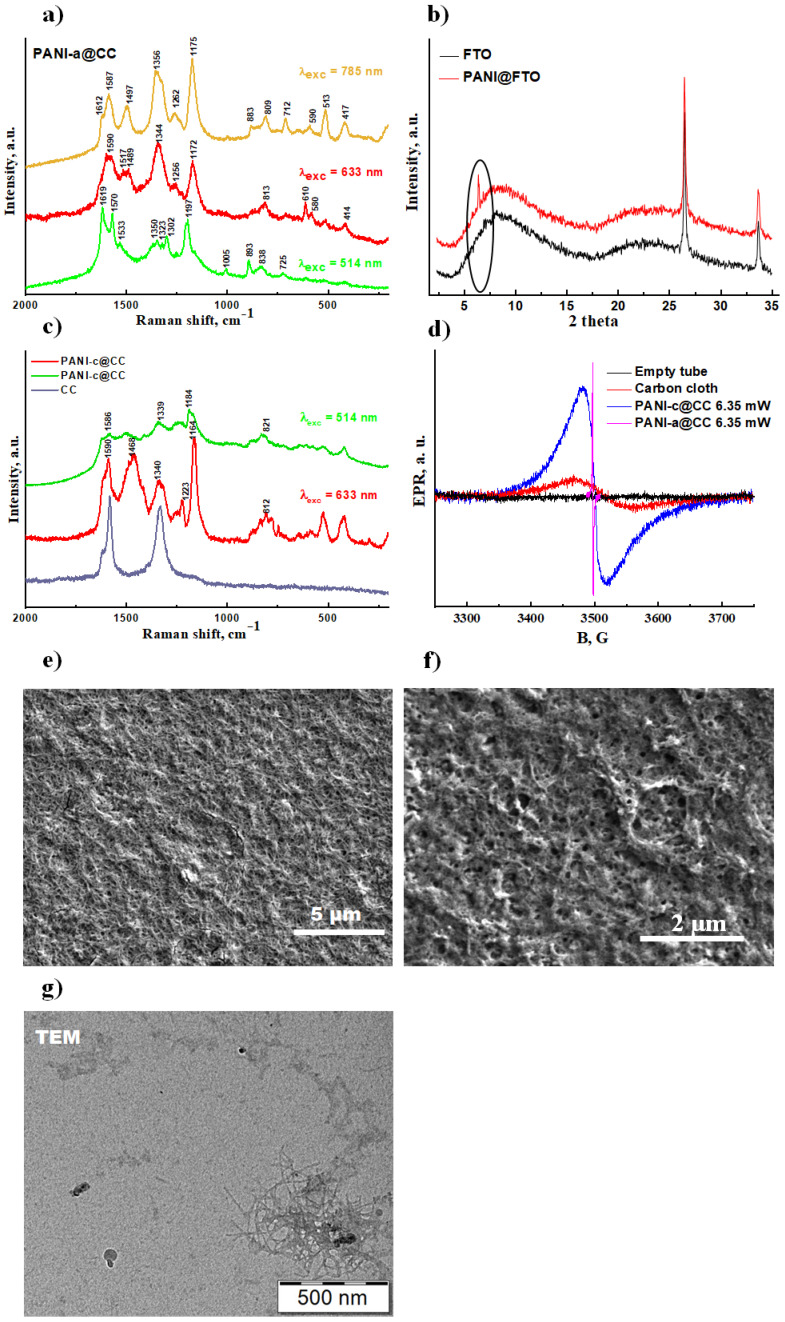
(**a**) Raman spectra of **PANI-a** on the carbon cloth (**PANI-a@CC**) recorded with excitation lines of 514, 633, and 785 nm; (**b**) XRD data for **PANI-a** deposited on FTO glass (**PANI-a@FTO)**; (**c**) Raman spectra of **PANI-c** on the carbon cloth (**PANI-c@CC**) recorded with excitation lines of 514 and 633 nm; (**d**) Normalized EPR signal—a first derivative of resonant microwave absorption (arbitrary units) vs. the external magnetic field for **PANI-a@CC and PANI-c@CC**; (**e**,**f**) SEM of **PANI-a@FTO** film with different magnifications; (**g**) TEM of **PANI-a** suspension.

Raman spectra were measured with excitation lines of 785 and 633 nm, and spectra for both **PANI-a@CC** and **PANI-c@CC** were found to be identical regardless of the excitation laser. Figure 2a shows the Raman spectrum for **PANI-a@CC**, while the spectrum of **PANI-c@CC** is presented in Figure 2c for comparison. The position and intensities of the main peaks in **PANI-a@CC** confirmed the formation of polyaniline at the surface of the CC (Figure 2a). The band at 1590 cm^–1^ was assigned to the C=C stretching vibration and the band at 1500 cm^–1^—to the C=N stretching vibration in the quinoid ring. The band linked with the C stretching vibration of the C~N^+^⋅delocalized polaron structure was observed at 1350 cm^–1^. The relatively high intensity was previously ascribed to the formation of charge transfer between PANI and carbonaceous material [46]. The characteristic band for C–N stretching in benzene rings was found at 1260 cm^–1^. The sharp intensive peak at 1175 cm^–1^ was attributed to the deformation C-H vibrations in the semi-quinoid rings. The band at 810 cm^–1^ was assigned to the ring deformation vibration of the protonated structures [46,47]. As the Raman excitation line of 785 and 633 nm is known to be in resonance with the quinoid units of PANI [48], additional Raman spectrum was measured with the excitation line of 514 nm (Figure 2a). In contrast to spectra recorded with the 785 and 633 nm excitation lasers, the band associated with the C~C vibrations in the benzenoid rings at 1619 cm^–1^ substantially shifted and its intensity significantly increased when spectrum was measured with the excitation 514 nm line. The novel band associated with the ring vibrations of the quinoid units appeared at 1570 cm^–1^. Another new band at 1350 cm^−1^ was subsequently assigned to the delocalization of the polaron in the C~N^+^⋅structure. The band corresponding to the vibration of secondary amine was observed at 1302 cm^–1^_._ Finally, the band at 1197 cm^–1^ was assigned to the C–H deformation vibrations in the rings [48,49]. Based on the Raman data analysis, it can be concluded that **PANI-a** has a chemical structure similar to **PANI-c**, although the mechanisms of the synthesis are different.

#### 3.3.2. X-ray Diffraction Studies

The XRD analysis for **PANI-a** was performed on the film deposited on FTO glass (**PANI-a@FTO**) and the result is shown in Figure 2b. The obtained diffraction patterns revealed broad humps at 2Θ = 9° and 23°, as well as two intense peaks at 2Θ = 26.51° and 33.71° (corresponding to (110) and (101) planes, respectively), originated from the FTO substrate [50]. The difference in the XRD patterns consisted of a sharp peak at 2Θ = 6.41° and was attributed to neat **PANI-a** (Figure 2b). According to the literature [51,52], this peak corresponds to interplanar spacing *d* = 13.8 Å and is associated with the distance between two stacks in the **2D stacking arrangement** of polymer chains created by the intervening dopant ions. Another difference relates to a slight increase in intensity of a broad reflection at about 2Θ = 22°, which is characteristic for amorphous PANI films and is attributed to the scattering from polymer chains due to π-π stacking interactions [53].

In contrast to **PANI-a**, the XRD pattern of **PANI-c** on the FTO glass exhibited several diffraction peaks [16,54]. The position of these peaks indicates the strong interchain packing, and clearly defined scattering refers to high crystallinity of the film [44]. The indexation of the respective reflections has been performed before by various authors (see for example [31]). Analyzing the XRD data, it can be concluded that **PANI-a** has 2D stacking arrangement of polymer chains together with the amorphous phase, in contrast to **PANI-c**, which possesses a high degree of crystallinity (up to 70%) [19].

#### 3.3.3. Electronic Paramagnetic Resonance (EPR)

To reveal further difference in the physical properties of **PANI-a** and **PANI-c** polymers, they were deposited on the conductive carbon cloth (CC) and the electronic paramagnetic resonance (EPR) was performed (Figure 2d). In the selected recording conditions, the resonator with an empty tube showed no signal. The neat CC showed a weak EPR Dyson line (ΔB_PANI-a_ = 73.7 G, A/|B| = 1.6, b = 0.23) and it was the main reason for the decrease in the resonator’s Q-factor. The **PANI-a@CC** demonstrated very narrow linewidth (ΔB_PANI-a_ = 0.87 G), large line asymmetry (A/|B|), which for multiple samples varied in the range 1.33–1.43, and g-factor equal to 2.0009 ± 0.0005. In comparison with **PANI-a@CC**, the **PANI-c@CC** showed a significantly wider linewidth ΔB_PANI-c_ = 34.7 G, smaller line asymmetry A/|B| in the range 1.08–1.34, b = 0.11, and nearly the same g-factor equal to 2.0003 ± 0.0005 (Figure 2d and Figures S2–S7). As both **PANI-a** and **PANI-c** polymers are conductive to the line asymmetry, A/|B| started to appear in the spectrum. This asymmetry is considered to be typical for conductive polymers where the gradient of microwaves penetrates the skin depth of the sample due to the skin effect. This results in the asymmetrical shape of the Dyson line detected in EPR spectroscopy. The asymmetry ratio A/|B| depends on specific conductivity and on the thickness and homogeneity of the sample on the CC. For two samples exhibiting the same specific conductivity, the thicker and more homogeneous polymer layer will cause larger line asymmetry [55,56]. The equal values of the g-factors found for both **PANI-a@CC** and **PANI-c@CC** samples are typical for materials that have free radicals and/or conductive electrons. The largest difference between the two polymers was observed in the linewidth, **which differed 40 times**. It is of common knowledge [57] that the line width is inversely proportional to the relaxation times ($\frac{1}{T_{2}^{\prime }}\sim \Delta B$), which in its turn allows for the assumption that the dissipation of microwave energy is faster for **PANI-c** polymer and the saturation effects will appear for higher microwave powers (Figures S3–S7). Thus, the EPR results are in good agreement with the measured XRD data (Figure 2b).

It is important to note that CC is visible in the background of the **PANI-c** spectrum for samples with minor deposition of the **PANI-c** polymer. The amplitude ratio (**PANI-c**/CC) is 6.3:1, the intensity ratio is ~3.3:1, and the linewidth ratio is ~2.1:1. In contrast to **PANI-c**, the CC signal in **PANI-a** sample was not detectable at all (Figure 2d). The Dyson lines were fitted with modified Dyson line [58] equations (Equations (1)–(5)):

(1) $$y=A\cdot \left(f_{1}+f_{2}\right)+y_{0}$$

(2) $$f_{1}=\frac{\left(b\cdot \left(1-p^{2}\right)-2p\right)}{{\left(1+p^{2}\right)}^{2}}\;\;$$

(3) $$\;f_{2}=\frac{\left(b\cdot \left(1-q^{2}\right)-2q\right)}{{\left(1+q^{2}\right)}^{2}}$$

(4) $$p=\frac{\left(x-x_{0}\right)}{w}$$

(5) $$q=\frac{\left(x+x_{0}\right)}{w}$$

where *A* is the amplitude, *w*—linewidth, *b*—function defined asymmetry parameter (0 is assigned for symmetrical parameter), *x*_0—_resonance field, *y*_0_—constant (Table 2).

#### 3.3.4. Scanning (SEM) and Transmission Electron (TEM) Microscopies

The microstructure of **PANI-a** was examined via scanning electron microscopy (SEM) on the FTO glass slides (Figure 2e,f). According to the SEM image, the **PANI-a** has nanofibrillar morphology similar to that reported for **PANI-c** [44], with higher porosity. The size of nanofibrillar can be estimated as 200 nm–1 μm. The observed porosity will play a crucial role in the impregnation of **PANI-a** film with solid polymer electrolyte, as it will be shown during the formation of a cathode material (see Section 3.5 below).

The transmission electron analysis (TEM) was carried out for **PANI-a** suspension on Pt-grid (Figure 2g). The TEM image clearly demonstrates the formation of the nanofibrils. Therefore, it can be concluded that during the suggested acid-assisted polymerization in highly concentrated acidic medium the synthesized PANI chains are assembled into 2D structures with nanofibrillar morphology. The size of the latter according to TEM analysis can be estimated as 100–150 nm, which is lower than that determined by SEM (200 nm–1 μm) and higher than that calculated with the results of DLS (50 nm, see Section 3.2). The difference in the nano-objects size between these methods can be driven by different sample preparations: while the DLS/SLS measurements were performed using the as prepared or diluted suspensions, the SEM/TEM analysis was conducted for the **PANI-a** films, where the particles were agglomerating and collapsing during the casting and drying process. In DLS, we measured swollen particles directly in the suspension and, therefore, DLS is a more precise method for determining the particle size of **PANI-a** as it measures the particles in their natural state close to the solution.

#### 3.3.5. Cyclic Voltammetry (CV) and Electrochemical Impedance Spectroscopy (EIS)

**Figure 3:**
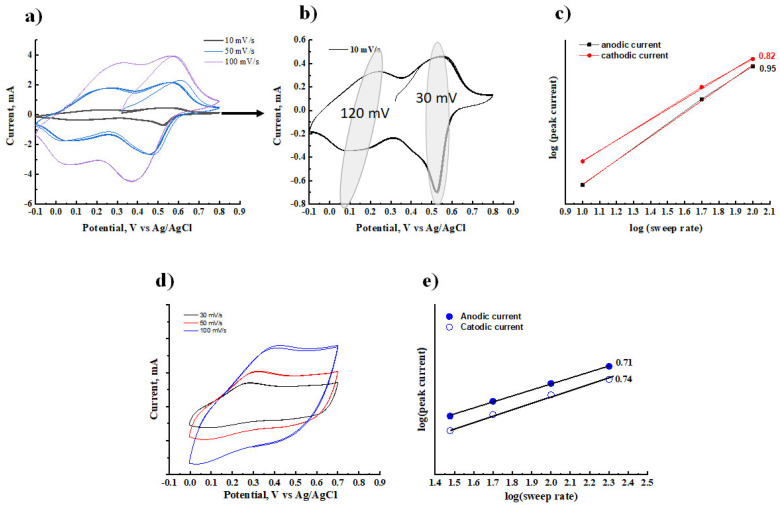
(**a**) Cyclic voltammetry of **PANI-a@CC** measured with different scan rates in 0.2 M aq. HCl electrolyte using the three-electrode cell configuration at 25 °C; (**b**) Detailed CV of **PANI-a@CC** at 10 mV s^−1^ scan rate; (**c**) Dependence of the log(sweep rate) for **PANI-a@CC**; (**d**) Cyclic voltammetry of **PANI-c@CC** measured with different scan rates in 0.2 M aq. HCl in three-electrode cell configuration at 25 °C; (**e**) Dependence of the log(sweep rate) for **PANI-c@CC**.

The electrochemical performance of the **PANI-a@CC** was first investigated by cyclic voltammetry, varying the scan rates in the range from 10 to 100 mV s^−1^, and was further compared with the data obtained for **PANI-c@CC** (Figure 3). It is important to note that **PANI-a** film was deposited with the polymer mass loading of 30 mg cm^−2^, while **PANI-c** had only 2.5 mg cm^−2^ mass loading. As discussed above, this difference in the mass loading is connected with the limitations of each synthesis/deposition method. The **PANI-a@CC** film clearly demonstrated the symmetrical cyclic voltammetry curves independently of the scan rate applied (Figure 3a,b). The CV curves of **PANI-a@CC** demonstrated the presence of two oxidations and two reductions peaks that we found to be revisable. The cyclic voltammetry measurements for **PANI-a@CC** revealed that the separation between anodic and cathodic peaks is equal to 120 mV for the first pair and 30 mV for the second pair (Figure 3b). Moreover, the deposited high mass loading of **PANI-a** has dependences of the log (peak current) for the anodic and cathodic currents vs. the log (sweep rate), with their slopes approaching unity (Figure 3c).

According to previous investigations, the shape of the EAECPs cyclic voltammograms, the position, and the number of the redox peaks were found to be influenced by the macromolecular arrangements, and more importantly by the interactions between polymer chains (see [16,44]). As discussed above, the XRD data (Figure 2b) showed that **PANI-a** possesses 2D structure incorporated into the amorphous phase that can clearly explain its unique electrochemical performance despite the high polymer mass loading. In contrast to **PANI-a**, the **PANI-c@CC** film, despite its significantly lower polymer mass loading (2.5 mg cm^−2^), showed the dependences of the log (peak current) for the anodic and cathodic currents vs. the log (sweep rate) with the slopes of 0.71 and 0.74, respectively (Figure 3e). Moreover, the oxidations and reductions peaks observed for **PANI-a@CC** were absent on the CV curves of **PANI-c@CC**, having had an almost rectangular shape (Figure 3a,d). It can be concluded that the simultaneous possession of 2D organization and amorphous phase in **PANI-a** leads to faster oxidation and reduction in comparison with **PANI-c**, having a compact crystalline-like structure. The calculated slopes of the log (peak current) vs. the log (sweep rate) dependence additionally prove that the intercalation (diffusion) of ions into the **PANI-a** film is happening much faster than in the **PANI-c** electrode (Figure 3c,e).

### 3.4. Synthesis and Characterization of Solid Polymer Electrolyte (SPE)

**Scheme 3:**
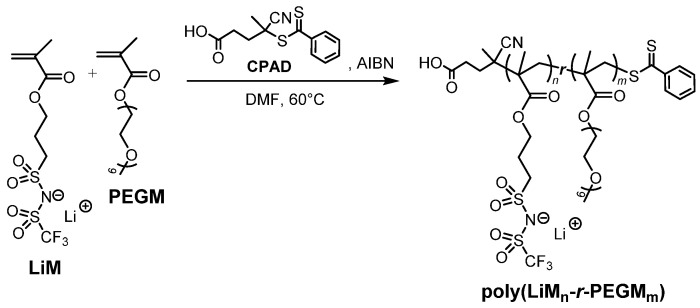
Synthesis of poly(LiM-*r*-PEGM) solid polymer electrolyte.

Copolymerization of lithium 1-[3-(methacryloyloxy)propylsulfonyl]-1-(trifluoromethanesulfonyl)imide (LiM) with poly(ethylene glycol)methyl ether methacrylate (Scheme 3) was performed via controlled reversible addition fragmentation chain-transfer polymerization (RAFT) using AIBN and 4-cyano-4-(phenylcarbonothioylthio)pentanoic acid (CPAD) as an initiator and chain transfer agent, respectively [22,59]. Synthesis was carried out with the conversion of 96%, as determined by H^1^ NMR, and resulted in the preparation of poly(LiM_13_-*r*-PEGM_81_) having *M*_n(SEC)_ = 43700 Da and satisfactory *M*_w_/*M*_n_ ratio (1.53). The composition, purity, and chemical structure of the obtained copolymer were supported by ^1^H, ^19^F, ^7^Li NMR, and FTIR spectroscopies. The isolated poly(LiM_13_-*r*-PEGM_81_) represented pink highly viscous cold flowing liquid with a low glass transition temperature equal to −53 °C, as determined by DSC. The investigation of its ionic conductivity in anhydrous conditions provided σ_DC_ value of 4.1 × 10^−7^ S cm^−1^ at 25 °C. The electrochemical stability window of the poly(LiM_13_-*r*-PEGM_81_) copolymer was studied by cyclic voltamperometry at 70 °C in Li||polymer||Cu configuration and the results are presented in Figure S8. The ESW of poly(LiM_13_-*r*-PEGM_81_) was found to be 4.3 V versus Li^+^/Li.

### 3.5. Composite PANI-a/SPE Cathode Material: Preparation and Testing

**Figure 4:**
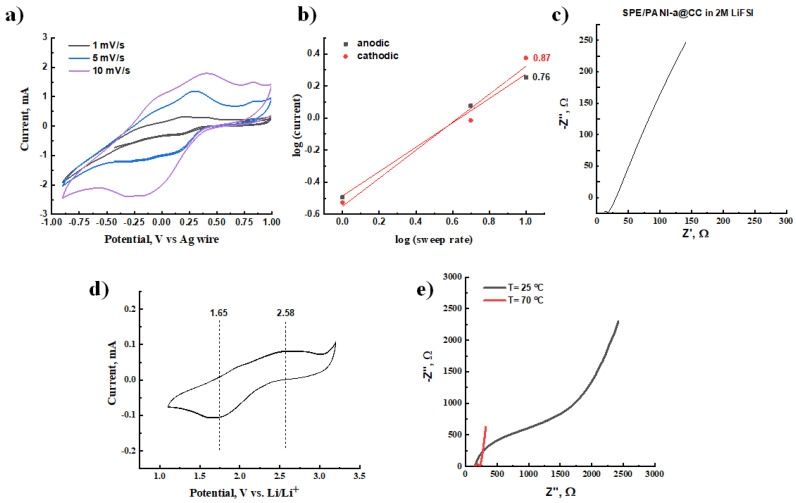
(**a**) Cyclic voltammetry of **SPE/PANI-a@CC** electrode at different scan rates in 2 M LiTFSI solution in a mixture of ethylene carbonate/dimethyl carbonate (1:1 by volume) at 25 °C; (**b**) Dependence of the log(sweep rate) for **SPE/PANI-a@CC** electrode; (**c**) electrochemical impedance spectroscopy of **SPE/PANI-a@CC** electrode measured at open circuit potential at 25 °C in 2 M LiTFSI solution in a mixture of ethylene carbonate/dimethyl carbonate (1:1 by volume) at 25 °C; (**d**) Cyclic voltammetry of **SPE/PANI-a@CC** in Li||**SPE/PANI-a@CC**||Cu configuration cell at 1 mV s^−1^ at 70 °C; (**e**) electrochemical impedance spectroscopy of **SPE/PANI-a@CC** electrode at 25 and 70 °C, measured in a solid state between two steel plates.

The cyclic voltammetry measurements of **PANI-a@CC** clearly demonstrated that electrochemical processes were not limited by diffusion, even when thick **PANI-a** film with a high mass loading of polymer was tested. This observation opened up possibilities to apply **PANI-a** not only as a promoter of electronic conductivity in addition to common inorganic LFP or NMC materials, but also as an original complete cathode material. Moreover, with the aim to enhance safety and to create an all-polymer-based battery, the **PANI-a@CC** was further combined with ionically conductive poly(LiM_13_-*r*-PEGM_81_). The impregnation of the single-ion conducting solid polymer electrolyte (SPE) was performed through the deposition of its NMP solution on the surface of **PANI-a@CC** with subsequent drying. The unique porous 2D stacking arrangement of **PANI-a** polymer chains allowed for the perfect penetration of SPE macromolecules and their mutual intercalation inside the **SPE/PANI-a@CC** electrode. This novel cathode material was tested by cyclic voltammetry and electrochemical impedance spectroscopy in 3-electrode cell configuration, wherein the results are presented in Figure 4a.

The CV examination of **SPE/PANI-a@CC** electrode was performed in 3-electrode configuration, using 2 M LiTFSI solution in a mixture of ethylene carbonate/dimethyl carbonate (1:1 by volume) as an electrolyte (Figure 4a). Moreover, the LiTFSI containing electrolyte was chosen with additional purpose to demonstrate the high levels of accessibility of the **SPE/PANI-a** layer to Li^+^ ions insertion/diffusion. Due to the nature of the selected liquid electrolyte, it was possible to increase the limits of the potential window, and the testing was carried out in the range from −0.9 V to 1 V vs. pseudo-reference Ag wire electrode. Several scan rates from 1 to 10 mV s^−1^ were applied to reveal the level of SPE and **PANI-a** intercalation. It was observed that both oxidation and reduction currents were increased proportionally with the acceleration of the scan rate, which can serve as a proof perfect SPE impregnation and that no SPE layer was blocking the surface of the PANI network. Moreover, the dependencies of the log (peak current) for the anodic and cathodic currents vs. the log (sweep rate) decreased slightly (0.76 for anodic and 0.87 cathodic, see Figure 4b) compared to the electrodes without SPE (Figure 3c). It can be concluded that the impregnation of PANI via drop casting of SPE solution did not block the surface of PANI. However, the impregnation/deposition process can still be optimized to fully utilize the electrochemical performance of PANI.

The charge transfer resistance, which is defined as a measure of the difficulty encountered when an electron is shifted from one atom in PANI macromolecule to another atom or to current collector, was estimated for **SPE/PANI-a@CC** electrode by electrochemical impedance spectroscopy (Figure 4c). In the **SPE/PANI-a@CC** composite cathode, the charge transfer resistance can happen between the SPE and PANI or PANI and CC interfaces. The measurement was performed at open circuit potential and at 25 °C. Figure 4c clearly demonstrates that the EIS curve does not have any semi-circle, which is the characteristic feature of the charge transfer resistance between PANI/SPE. Such a shape allows us to conclude that the newly obtained **PANI-a** structure/morphology is ideal for its impregnation by the solution of a single ion conducting polyelectrolyte and leads to the formation of an attractive composite cathode material with low charge transfer resistance.

To ensure the ability of the **SPE/PANI-a@CC** composite cathode to work with Li metal, the cyclic voltammetry of a Li||**SPE/PANI-a@CC**||Cu cell was studied at 70 °C (Figure 4d). The CV showed two broad symmetrical peaks at 2.58 and 1.65 V vs. Li/Li^+^. These peaks correspond to oxidation and reduction reactions, respectively. The maintenance of their shape and position upon cycling (5 continuous cycles) clearly demonstrates that the doping/de-doping processes are reversible [60]. The total obtained ESW of 2.1 V vs. Li/Li^+^ fully coincided with the stability window (2.4 V vs. Li/Li^+^) of a cathode composed of perchloricacid-doped PANI nanotubes [13]. To further show the potential of the **SPE/PANI-a@CC** cathode to work in all-solid-state batteries, its impedance was measured between two stainless steel plates in the absence of any liquid electrolyte at 25 and 70 °C (Figure 4e). The temperatures of experiment were selected by the following reasons: (a) solid state polymer Li batteries typically operate at elevated temperatures (70 °C); (b) given the unstable nature of many conducting polymers, it is important to demonstrate that PANI provides stable performance at elevated temperatures (70 °C) required by batteries. The total impedance of the **SPE/PANI-a@CC** in a solid state at 25 °C showed an increase in comparison with the measurement performed for the same material in 2 M LiTFSI liquid electrolyte (Figure 4c). However, this can be leveled by an increase in temperature to 70 °C and the enhancement of the ion’s mobility and conductivity in SPE, leading to a decrease in both the real and imaginary parts of the impedance spectra (Figure 4e).

## 4. Conclusions

A novel method for the synthesis of polyaniline (PANI) via acid-assisted polymerization in concentration solution has been proposed for the first time. The physical and electrochemical properties of the obtained **PANI-a** were studied in detail and compared with the **PANI-c** prepared by the well-known oxidative polymerization procedure. The most striking advantages of **PANI-a** can be summarized as follows: (1) the polymer is formed in a form of stable suspension that consists of rod-like particles with a size of 50 nm and stabilized by the formation of the positively charged form via the protonation with concentrated formic acid; (2) the deposition of the concentrated PANI-a suspension of FTO glass slides or carbon cloth surface results in *never-before-achieved record high mass loading of electro-active material* (30 mg cm^−2^); (3) the deposited **PANI-a** chains are amorphous and assembled into 2D structures with nanofibrillar morphology; (4) the **PANI-a** film shows very fast and efficient diffusion of the ions through its body, as proved by the presence of two revisable oxidation and reduction peaks in cyclic voltammetry; (5) the morphology and porosity of **PANI-a** film allows for its impregnation with the solid polymer electrolytes and leads to the formation of all-polymer-based cathode materials with excellent electrochemical performance and a promising capacity for future application in supercapacitors and batteries.

## Figures and Tables

**Table 1:** SLS and DLS results of **PANI-a** suspension in formic acid.

*R*_H,0_ (nm) ^1^	*R*_g,app_ (nm)	*M*_w app_ × 10^8^ (g mol^−1^)	*D*_app_ × 10^15^ (cm^2^ s^−1^)	*v*
56.8 ± 11.4	97.6 ± 19.5	7.3 ± 1.5	2.89 ± 0.075	1.72

^1^ *R*_H,0_ is the hydrodynamic radius at zero angle, *R*_g,app_ is the radius of gyration of rod-type particles, *M*_w app_ is the weight average particles molecular weight, and *D*_app_ is the collective diffusion coefficient, calculated as the slope angle of the curve in Figure 1.

**Table 2:** Fitting parameters with modified Dyson line.

Sample	A, (arb. u.)	w, (G)	b, (abr. u.)	A/|B|	x_0_, (G)
Carbon cloth	0.16	73.7	0.33	1.6	3503.9
**PANI-c**	1.19	34.7	0.11	1.08–1.34	3497.9
**PANI-a**	14.10	0.87	0.23	1.33–1.43	3497.4

## Data Availability

The data presented in this study are available on request from the corresponding author.

## Supplementary Materials

The following supporting information can be downloaded at: https://www.mdpi.com/article/10.3390/polym15112508/s1, Figure S1: Normalized EPR signal of polymerization mixture (**PANI-a**) immediately after preparation (t = 0) and after 24 h; Video S1: synthesis of PANI suspension by acid-assisted polymerization; Figure S2: EPR spectra of carbon cloth used as support for polymer synthesis and empty tube; Figure S3: Microwave room temperature saturation experiment of **PANI-a@CC** and **PANI-c@CC** polymer; Figure S4: EPR spectra of **PANI-c** polymer covering carbon cloth, carbon cloth recorded separately and the difference spectrum of only **PANI-c** polymer without carbon cloth contribution. Figure S5: EPR line of carbon cloth and the fit with modified Dyson line; Figure S6: EPR line of **PANI-c** polymer after subtraction of carbon cloth and the fit with modified Dyson line; Figure S7. EPR line of **PANI-a** polymer and the fit with modified Dyson line; Figure S8: Electrochemical stability windows obtained by CV for poly(LiM-r-PEGM) solid polymer electrolyte at 70 °C (stainless steel as a working electrode and Li foil as counter and reference electrodes, scan rate 0.2 mV s^−1^).
