# Supplementary material for: A New Method to Prepare Stable Polyaniline Dispersions for Highly Loaded Cathodes of All-Polymer Li-Ion Batteries
Source: Polymers (Basel). 2023 May 29;15(11):2508. doi: 10.3390/polym15112508 (PMC10255123; doi:10.3390/polym15112508)
Supplement: Supplementary file 1 [file polymers-15-02508-s001.zip › polymers-2324230-supplementary.pdf]

Supporting Information

Article

A New Method to Prepare Stable Polyaniline Dispersions for Highly Loaded Cathodes of All-Polymer Li-Ion Batteries

Elena Tomšík [1,*,†], Daniil R. Nosov [2,3,†], Iryna Ivanko [1], Václav Pokorný [1], Magdalena Konefał [1], Zulfiya Černochová [1], Krzysztof Tadyszak [1], Daniel F. Schmidt [2] and Alexander S. Shaplov [2]

[1] Institute of Macromolecular Chemistry AS CR, Heyrovského Nám. 2, 162 00 Prague, Czech Republic; ivanko@imc.cas.cz (I.I.); pokorny@imc.cas.cz (V.P.); magdalenakonefal@imc.cas.cz (M.K.); cernochova@imc.cas.cz (Z.Č.); negaton83@gmail.com (K.T.)

[2] Luxembourg Institute of Science and Technology (LIST), 5 Avenue des Hauts-Fourneaux, L-4362 Esch-sur-Alzette, Luxembourg; daniil.nosov@list.lu (D.R.N.); daniel.schmidt@list.lu (D.F.S.); alexander.shaplov@list.lu (A.S.S.)

[3] Department of Physics and Materials Science, University of Luxembourg, 2 Avenue de l'Université, L-4365 Esch-sur-Alzette, Luxembourg

* Correspondence: tomsik@imc.cas.cz; Tel.: +420-296-809-351

† These authors contributed equally to this work.

Keywords: polyaniline, acid-assisted, energy, 2D materiál

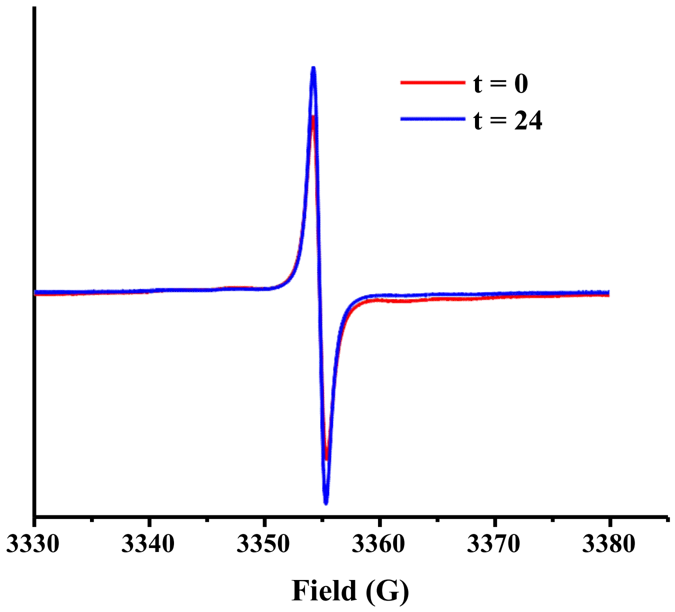

Figure S1. Normalized EPR signal of polymerization mixture (**PANI-a**) immediately after preparation (t = 0) and after 24 h.

IMG_2915.MOV

IMG_2920.MOV

Video S1. Synthesis of PANI suspension by acid-assisted polymerization.

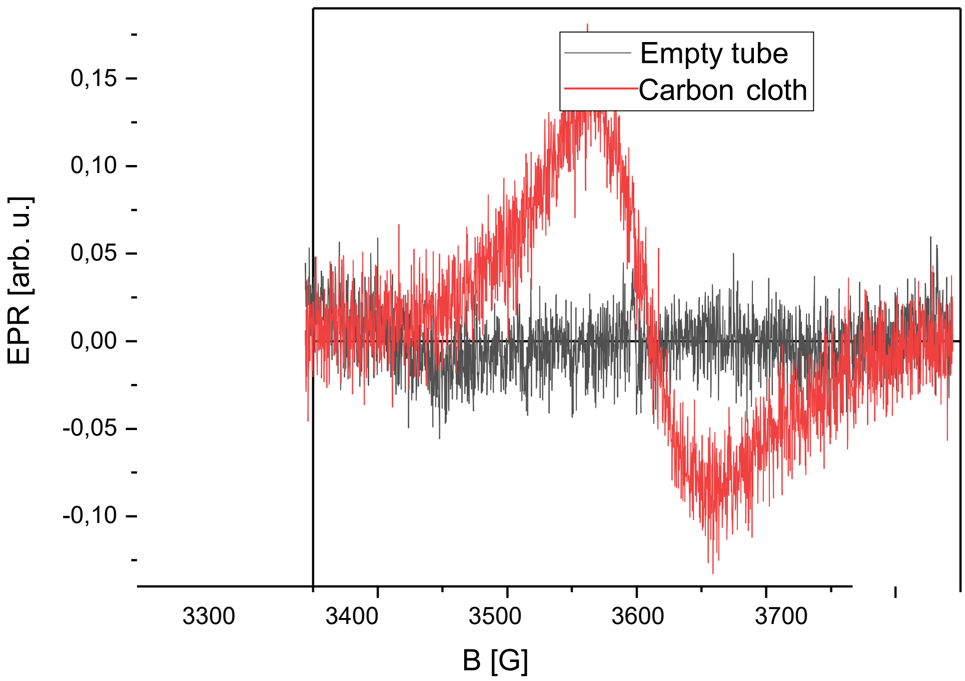

Figure S2. EPR spectra of Carbon cloth used as support for polymer synthesis (red), and empty tube (black).

The saturation experiment performed at 295 K (Fig. S4) is performed as follows. EPR spectrum is recorded with increasing microwave power to distinguish if the spectrum consists of multiple contributions in which relaxation times differ between them. If the criteria are met the line shape of the spectrum would have change by decreasing the line that relaxes slower and leaving uncharmed the line from the radical with faster relaxation time. Experiments performed for two polymers did not show any changes in the line shape and the relaxation time of both of them at room temperature was high enough to not be able to saturate the line with maximum power (200 mW).

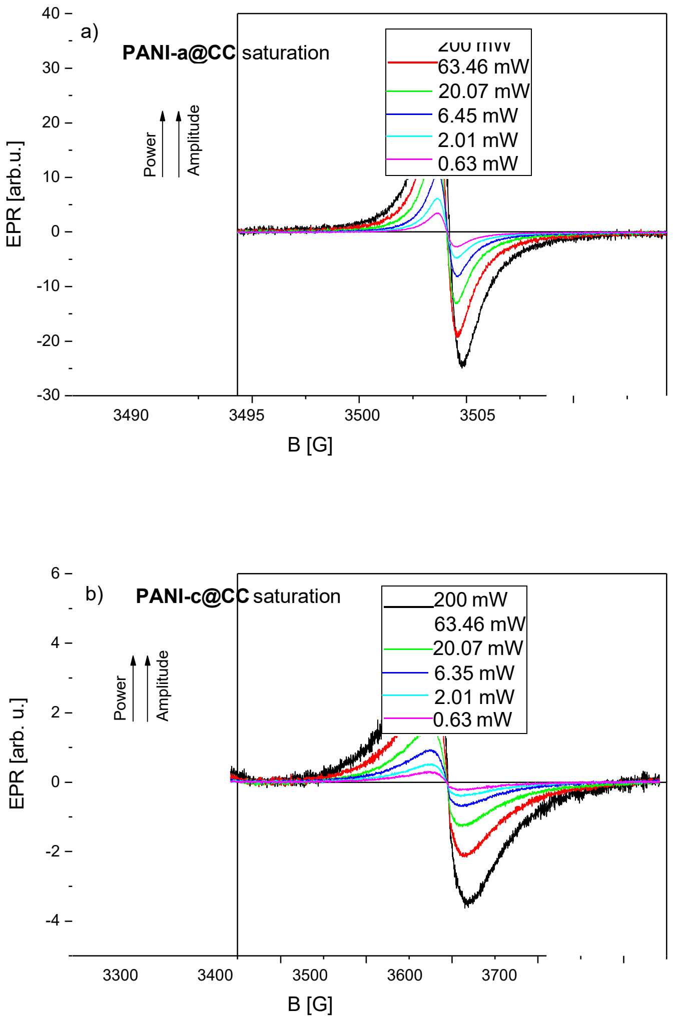

Figure S3. Microwave room temperature saturation experiment of a) **PANI-a@CC** and b) **PANI-c@CC** polymer.

Carbon cloth material used in electrochemical experiment as electrode on which polymers form a layer has shown its own weak and broad signal. In case of the polymer **PANI-c@CC** the contribution of the carbon cloth material was compared and substituted (**Fig. S5**). While measuring other **PANI-c@CC** samples which had more polymer deposited on its surface the carbon cloth signal is not detectable. In any case it was undetectable for **PANI-a@CC** polymer.

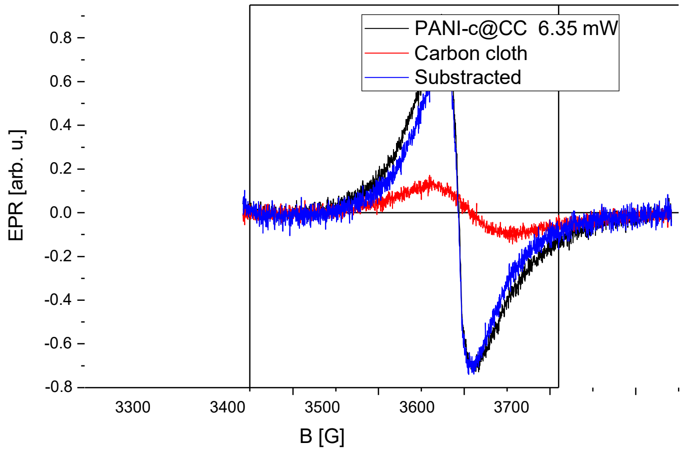

Figure S4. EPR spectra of **PANI-c** polymer covering carbon cloth (black), carbon cloth recorded separately (red), difference spectrum of only **PANI-c** polymer without carbon cloth contribution.

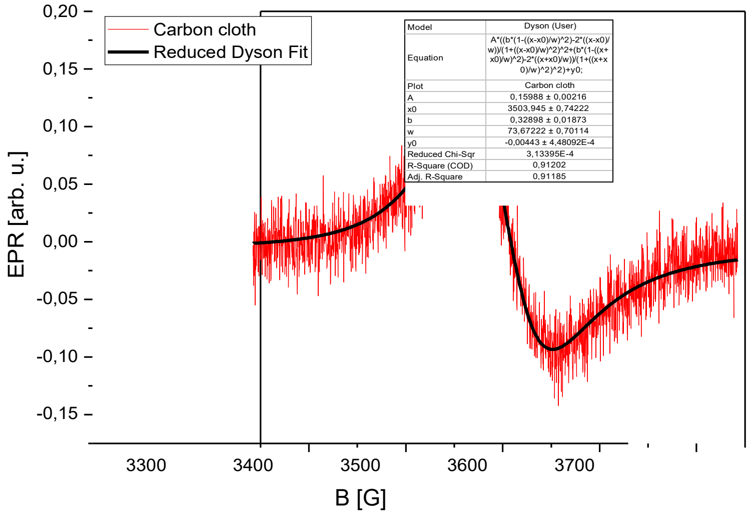

Figure S5. EPR line of carbon cloth (red, from Figure S4); fit with modified Dyson line.

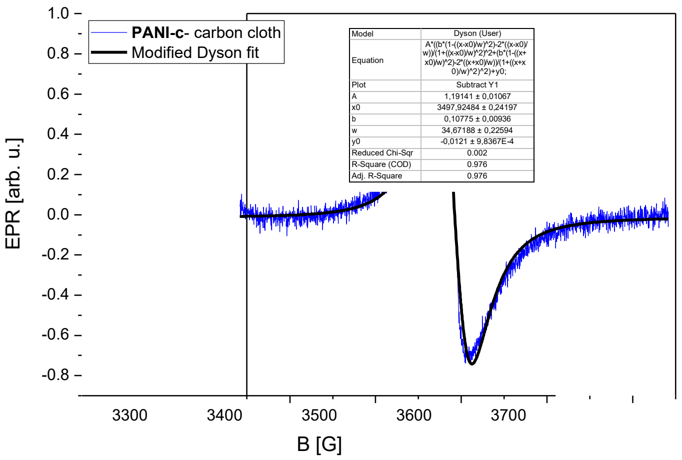

Figure S6. EPR line of **PANI-c** polymer after subtraction of carbon cloth (blue, from Figure S4); fit with modified Dyson line.

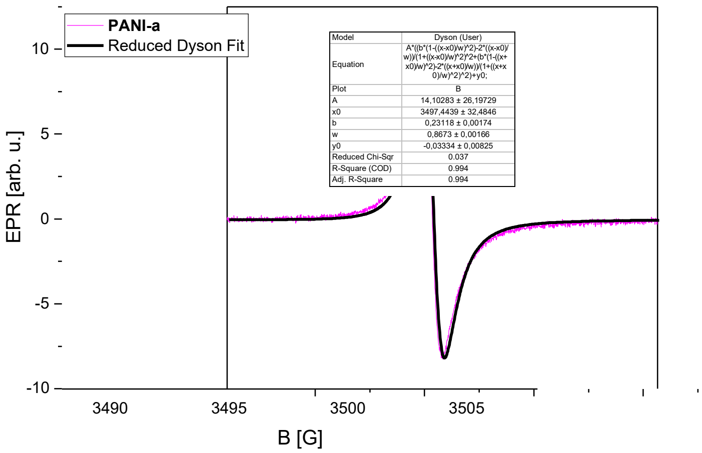

Figure S7. EPR line of **PANI-a** polymer (magenta, from Figure 2e); fit with modified Dyson line.

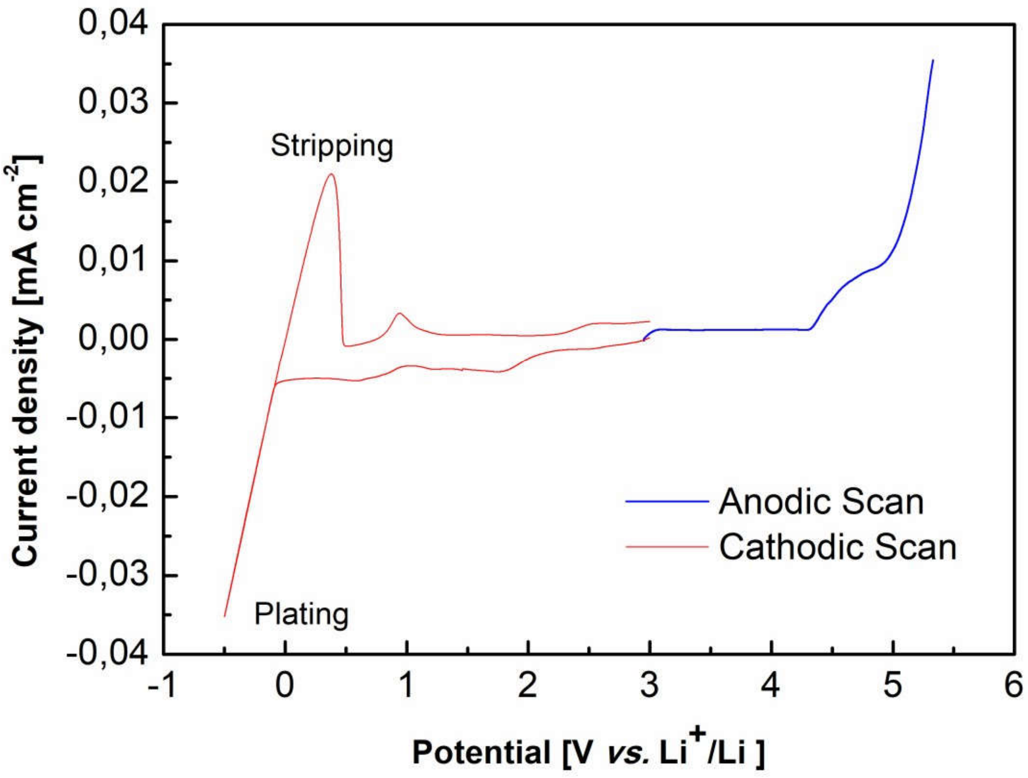

Figure S8. Electrochemical stability windows obtained by CV for poly(ILM-r-PEGM) solid polymer electrolyte at 70ºC (stainless steel as a working electrode and Li foil as counter and reference electrodes, scan rate 0.2 mV/s).

References

1. Dyson, F. J. Electron Spin Resonance Absorption in Metals. II. Theory of Electron Diffusion and the Skin Effect. *Phys. Rev.* **98**, 349–359 (1955).

2. Tadyszak, K., Strzelczyk, R., Coy, E., Mac'Kowiak, M. & Augustyniak-Jabłokow, M. A. Size effects in the conduction electron spin resonance of anthracite and higher anthraxolite. *Magn. Reson. Chem.* **54**, 239–245 (2016).

3. Wertz, J. E. & Bolton, J. R. *Electron Spin Resonance*. (Springer Netherlands, 1986). doi:10.1007/978-94-009-4075-8.

4. Popovych, V., Bester, M., Stefaniuk, I. & Kuzma, M. Dyson line and modified Dyson line in the EPR measurements. *Nukleonika* **60**, 385–388 (2015).
